# Stigma and low vision. An analysis of experiences and feelings

**DOI:** 10.15649/cuidarte.3974

**Published:** 2024-11-19

**Authors:** Diana Cristina Palencia-Flórez, María del Pilar Oviedo-Cáceres

**Affiliations:** 1 Universidad Santo Tomás, Bucaramanga, Colombia. E-mail: diana.palencia@ustabuca.edu.co Universidad Santo Tomás Universidad Santo Tomás Bucaramanga Colombia diana.palencia@ustabuca.edu.co; 2 Universidad Santo Tomás, Bucaramanga, Colombia. E-mail: maira.oviedo@ustabuca.edu.co Universidad Santo Tomás Universidad Santo Tomás Bucaramanga Colombia maira.oviedo@ustabuca.edu.co

**Keywords:** Social Stigma, Stereotyping, Self Concept, Quality of Life, Mental Health., Estigma Social, Estereotipo, Autoconcepto, Calidad de Vida, Salud Mental, Estigma Social, Estereotipagem, Autoimagem, Qualidade de Vida, Saúde Mental

## Abstract

**Introduction:**

Low vision is a sensory impairment that restricts the autonomous execution of activities of daily living, negatively affecting the quality of life and fostering the appearance of stigma.

**Objective:**

To explore the experiences and feelings about stigma experienced by people with low vision to provide elements that favor their understanding within the framework of a comprehensive care process.

**Materials and Methods:**

A phenomenological study was conducted, employing in-depth interviews with 10 low-vision individuals, selected based on convenience.

**Results:**

The reports indicate that the experiences associated with the stigmatization process include a lack of differentiation between individuals who are blind and those with low vision, inadequate signage, and cultural and communicative practices that are based on ocularcentrism. In terms of emotional responses, a lack of positive self-perception is the most significant factor, leading to social withdrawal. Finally, the proposed strategies compass the formation of support networks, the provision of psychosocial assistance, the adaptation of physical spaces, and the promotion of organizational restructuring in educational institutions and companies.

**Discussion:**

The stigma associated with low vision is the result of a set of cultural factors and stereotypes, which can be explained as a phenomenon through the socio-ecological model, providing information to design interventions at the individual, community, organizational, and structural levels.

**Conclusion:**

The experiences derived from social interaction, educational processes, and work activity generate feelings that modify the stigmatization process in low-vision individuals. Therefore, studying and planning health interventions from different levels is advisable, considering the gender and life cycle perspective.

## Resumen.

**Introducción:**

La baja visión es una discapacidad sensorial que restringe la ejecución autónoma de actividades de la vida diaria, lo que impacta negativamente la calidad de vida, y favorece la aparición del estigma.

**Objetivo:**

Explorar las experiencias y sentimientos en torno al estigma que viven las personas con baja visión para aportar elementos que favorezcan su comprensión en el marco de un proceso de atención sanitaria integral.

**Materiales y Métodos:**

Estudio fenomenológico, que empleó entrevistas a profundidad en 10 personas con baja visión seleccionadas a conveniencia.

**Resultados:**

Los relatos muestran que las experiencias asociadas al proceso de estigmatización comprenden: la falta de diferenciación entre ciegos y personas con baja visión, la escasa señalética, las prácticas culturales y comunicativas basadas en el ocularcentrismo. Respecto a los sentimientos, prima el pobre autoconcepto, que desencadena aislamiento social. Finalmente, se consideran estrategias el conformar redes de apoyo, brindar acompañamiento psicosocial; incentivar adecuaciones de los espacios físicos y promover la reestructuración organizacional en instituciones educativas y empresas.

**Discusión:**

El estigma asociado a la baja visión es el resultado de un conjunto de factores culturales y estereotipos, que puede ser explicado como un fenómeno a través del modelo socio-ecológico, que aporta información para diseñar intervenciones a nivel individual, comunitario, organizacional y estructural.

**Conclusión:**

En las personas con baja visión las experiencias derivadas de la interacción social, los procesos educativos y la actividad laboral generan sentimientos que modifican el proceso de estigmatización. Por ello, consideramos conveniente estudiar y planear las intervenciones de salud desde diferentes niveles, considerando a su vez la perspectiva de género y de ciclo vital.

## Resumo.

**Introdução:**

A baixa visão é uma deficiência sensorial que restringe a execução autônoma das atividades da vida diária, o que impacta negativamente a qualidade de vida e favorece o surgimento do estigma.

**Objetivo:**

Explorar as experiências e os sentimentos sobre o estigma vivenciados por pessoas com baixa visão a fim de fornecer elementos que favoreçam sua compreensão no âmbito de um processo de atenção integral à saúde.

**Materiais e Métodos:**

Estudo fenomenológico, por meio de entrevistas em profundidade com 10 pessoas com baixa visão selecionadas conforme sua conveniência.

**Resultados:**

Os relatos mostram que as experiências associadas ao processo de estigmatização incluem: a falta de diferenciação entre pessoas cegas e com baixa visão, sinalização deficiente, práticas culturais e comunicativas baseadas no ocularcentrismo. Com relação aos sentimentos, o autoconceito ruim, que desencadeia o isolamento social, é o mais importante. Por fim, as estratégias consideradas são a criação de redes de apoio, o fornecimento de apoio psicossocial, o incentivo à adaptação de espaços físicos e a promoção da reestruturação organizacional em instituições educacionais e empresas.

**Discussão:**

O estigma associado à baixa visão é o resultado de um conjunto de fatores culturais e estereótipos, que podem ser explicados como um fenômeno por meio do modelo socioecológico, que fornece informações para projetar intervenções nos níveis individual, comunitário, organizacional e estrutural.

**Conclusão:**

Nas pessoas com baixa visão, as experiências derivadas da interação social, dos processos educacionais e da atividade laboral geram sentimentos que modificam o processo de estigmatização. Portanto, consideramos conveniente estudar e planejar intervenções de saúde em diferentes níveis, considerando, por sua vez, a perspectiva de gênero e do ciclo de vida.

## Introduction

Stigma is a social and psychological phenomenon characterized by labeling, loss of social status, and discrimination associated with low vision, a type of sensory impairment characterized by a permanent and irreversible reduction in visual acuity, resulting in deficiencies in the perception of light, shape, size, or color of objects^1^.

According to global estimates, between 1990 and 2015, there was an increase of 35.5% in the number of cases of visual impairment, from 159.9 million to 216.6 million, figures that can be explained by factors such as population growth (38.48%), aging (29.25%), and greater availability of diagnostic alternatives (24.24%) and others (8.30%)^2^.

Figures are not the only challenge in managing low vision, as loss of visual function affects quality of life by causing delays in developing fine and gross motor skills. However, due to the difficulty in coordinating movements and exploring the environment, low vision also affects the development of cognitive skills such as visual memory and the recognition of objects or facial expressions and other non-verbal signals, which has an impact on social-emotional development^3^ and learning^2^. This accumulation of situations has implications for their continuing participation in the regular education system^4^, their access to formal employment^5^, and their ability to carry out activities of daily living^6^. In addition, in older adults, low vision increases the risk of injury, falls^7^, depression^8^, dependency, and early mortality^9^.

The impact of stigma on people with low vision can be explained by the fact that visual impairment imprints a differential characteristic in the collective imagination, which is configured as a discrediting attribute in the form of public stigma^10^. Indeed, individuals construct their identity and understand their environment through communication and interaction with others. In this way, they create self-labels that reinforce the cultural values of the dominant society, which are translated into a value judgment of negative connotation that ends up typifying disability as an "abnormal" or "different" condition, as described in labeling theory^11^. This typecasting favors the exacerbation of social isolation as a result of reduced participation in cultural and recreational activities; it also leads to increased exclusion, inaccessibility, and discrimination^12^.

In response to this situation, guidelines have been established, such as the United Nations Convention on the Rights of Persons with Disabilities, which has been in force since 2008^13^ and seeks to mitigate the barriers that seem to be perpetuated over time. Those of us who are interested in understanding the consequences of stigma for people with low vision believe it is necessary to explain the phenomenon from an integrative perspective. Specifically, we believe that models such as the socio-ecological model, which allows us to approach the stigmatization process from a series of levels ranging from the individual to the social and structural, would facilitate the understanding of the role of visual impairment in the context of health in Colombia, especially to identify multiple sources and levels of influence that promote stigmatization^14^.

In the Republic of Colombia, there are regulatory provisions, such as Law 1618 of 2013, which promote access to the fundamental principles of equality, non-discrimination, and respect for human dignity in the different human spheres^15^. However, public policy on visual impairment is still insufficiently regulated and lacks scientific evidence to support a strategic intervention plan^16^.

This normative and empirical vacuum, in addition to an individual who is aware of the stereotype, who adopts it and applies it himself, allows us to argue that internalized stigma or self-stigma is an urgent problem to be addressed from a reflective approach in which human experience is explored. We, therefore, see stigma and low vision as two dynamic and continuous processes that can be understood in their context through stories and narratives that are influenced by criteria of temporality, whose three-dimensional structure favors the understanding of the phenomena by considering the dimensions of past, present, and future^17^. Given the above, there is a need to explore the experiences and feelings related to the stigma experienced by people with low vision to provide elements that favor their understanding within the framework of a comprehensive healthcare process.

## Materials and Methods

A qualitative study was conducted using interpretive phenomenology to understand everyday experiences and articulate both similarities and differences in the meanings given to stigmatizing situations by people with low vision.

### Sample

Ten individuals with confirmed low vision based on their degree of visual impairment, visual field deficit, or reduced functionality in activities of daily living, who reported being engaged in work or educational activities within the past year, were interviewed. People with coexisting disabilities were excluded to ensure clarity of results, as differences in the reporting of experiences may arise when including people with multiple disabilities. People with psychiatric disorders were also excluded, as the literature documents specific forms of stigma representation for this type of population^18^. The researchers had no prior contact with the participants before the interviews.

The sample was selected by convenience from the list of people with visual impairment treated between April and June 2024 at an ophthalmological center in the city of Medellin and an optometry service at a university in Bucaramanga. Participants with complete clinical data, which allowed verification of the selection criteria, were included, as we wanted to collect information on the experiences of people with both congenital and acquired visual impairment of both sexes residing in two Colombian cities.

### Data collection

During the defined months for data collection, 24 patients were included in the low vision services of Medellin and Bucaramanga. Of these, 8 were excluded for not meeting the selection criteria, and 6 declined to participate (4 because they did not feel psychologically prepared to share their experiences and 2 due to lack of time to attend the interview). During the initial contact, the study was explained, the informed consent form was signed, and clinical information about the visual diagnosis was requested.

In the second contact, in-depth interviews of approximately one hour were conducted with each participant. Some interviews were held face-to-face, while others were technologically mediated through the Microsoft Teams® platform. An interview script was developed using Pinel's Stigma Consciousness Questionnaire^19^ as a reference, covering aspects such as experiences of feeling judged when interacting with others in family, work, or educational settings, as well as their perceptions of differential treatment due to their low vision condition. We also asked about the emotions and feelings that arise when interacting with others or entering a work or educational environment for the first time. In addition, information related to the visual diagnosis was taken into account when answering the questions.

A pilot interview was conducted with a patient who was not part of the final sample to assess the clarity, relevance, and receptiveness of the guiding questions. In the first part of the interview, we asked about participants' perceptions of stigma and disability status and allowed them to freely express their stigmatizing experiences in family, social, academic, and work environments. Finally, we discussed the feelings generated as a result of these experiences and the mechanisms they found beneficial in the process of coping with the stigma associated with low vision.

Interviews were recorded and transcribed verbatim. They were stopped according to the criterion of theoretical saturation^20^. Data analysis was conducted in parallel with data collection, beginning with a narrative content analysis. At the end of the data collection, a deep analysis was carried out, involving reading and rereading the interviews to identify the essential structure of both experiences and feelings, comparing what was reported in search of similarities and differences to identify the units of analysis. These units were then coded, and categories to be addressed were identified. The findings were reviewed with some participants to ensure that the descriptions accurately reflected the experiences, and a low-vision optometrist and a rehabilitation specialist were invited to review the findings.

The study was considered minimal-risk research according to Resolution 8430 of 1993 of the Colombian Ministry of Health and Social Protection and was approved by the Ethics Committee of the Santo Tomás University [Bucaramanga, Colombia]. A sequential code was assigned for identification during the transcription of the interviews. The data collected are freely available in Mendeley Data^21^.

## Results

Of the 10 participants, five men and five women aged between 22 and 50, six were from Medellín and four from Bucaramanga. Two were students at private universities and the rest were professionals in the fields of education and economic, administrative, and accounting sciences; some of them were working, and others had retired due to their disability.

By analyzing the information gathered within the framework of Pinel's theoretical approach, which promotes understanding of how people perceive, express, and cope with the stigma associated with their visual impairment by highlighting its psychological and social implications, three categories emerged. These categories reflect key elements in understanding stigma from the perspective of people with low vision, as outlined below:

### 1.Factors contributing to stigma

Specific factors within social contexts are identified as having deeply ingrained cultural roots that facilitate the consolidation of negative stereotypes about visual impairment. Compounding this is a lack of knowledge about the specificities of low vision, which leads to categorizing everyone as blind without distinguishing between total and partial vision loss, thus encouraging comments such as the following:

*“Getting a job as a person with a disability like me isn’t easy. People don't understand that I can do everything, even though it takes me a bit longer, and they often make comments like 'Oh, he only sees what suits him or when it suits him.’" (Male, 28 years old, congenital).*

The situation is exacerbated by the lack of comprehensive information during the medical care process, including delays in confirming the diagnosis, difficulties accessing periodic check-ups, and inadequate advice on rehabilitation alternatives. These challenges are reflected in comments such as:

*“I'd never been told about rehabilitation. In fact, it wasn’t until I started university that I found out I had low vision. I always just thought that I saw less than others, and that was it” (Female, 22, acquired).*

This demonstrates that coping with low vision, while not ideal, is feasible even without a structured rehabilitation program, particularly in developing countries where healthcare services are fragmented. In such contexts, individuals often resort to autonomous adaptation processes, which are further complicated by factors such as the limited use of signage, poor road safety culture, and the inadequate condition of sidewalks and public spaces.

In addition to physical barriers, it is also important to recognize that our society is based on traditional forms of communication characterized by vocalized speech and eye contact. These forms of interaction reinforced by the media contribute to a tendency toward ocularcentrism.

The phenomenon of ocularcentrism represents a persistent challenge for individuals with low vision, and unfortunately, individuals often avoid the use of support devices, whether optical or non-optical, due to the potential for these devices to reveal a disability that may not have been previously apparent. When such disabilities are identified, they can create a divide between those who experience low vision and the rest of society. In this regard, statements such as:

*“I try to hide the fact that I have low vision. That's why I've never used a cane. I don't want people to treat me differently” (Male, 28, acquired).*

In academic and professional settings, it is important to recognize that individuals with impaired visual function may face challenges in achieving goals within specific timeframes. These individuals often experience eye fatigue, photophobia, fluctuating vision, headaches, and other symptoms that can make it difficult to perform tasks. This situation can lead to form a priori judgments that reinforce identity devaluation.

### 2. Stigma manifestations

The lack of vision is typically perceived with a negative connotation, as individuals with "normal" vision often struggle to comprehend the reality of those living with visual impairment and often adopt discriminatory attitudes, which in turn influence the construction of self-concept and lead to the internalization of both prejudice and negative attitudes.

A poor self-concept is a significant risk factor for low self-esteem, which, in turn, increases the likelihood of social isolation and compromises mental health, as well as effectively accessing a rehabilitation process. It is even conceivable that individuals may choose to decline medical care and formal educational opportunities that facilitate access to decent working conditions. Such attitudes are exemplified by comments such as:

*“I used to think that I wouldn't be able to pursue a university degree or even hold a job” (Male, 38, acquired).*

It is also notable that individuals with low vision encounter additional barriers to promotion in formal employment settings. Their competencies and contributions are frequently underestimated.

### 3. Strategies to mitigate the effects of stigma

Different forms of stigma manifest in individuals with low vision, whether congenital or acquired. However, how stigma is taken varies considerably due to multiple factors, including the structure of the family unit and how its members respond to visual impairment, as well as cultural particularities that establish social standards.

Since low vision and stigma are converging constructs, reviewing strategies that facilitate management, such as creating support networks within familial, social, occupational, and even medical contexts is imperative. The formation of these networks provides opportunities to learn valuable lessons, such as:

*“When I talked to people who were in similar situations, I realized that things could be accomplished, that I could move forward and had a future ahead of me” (Female, 40, acquired).*

Psychosocial support is important to promote coping with disability:

*“As a kid, I would just go from home to school... I barely had any friends and thought there was something wrong with me. Later, I realized I wasn't the problem. I was simply someone with different abilities” (48-year-old man with congenital visual impairment).*

The implementation of mechanisms that prioritize comprehensive rehabilitation within a healthcare model that articulates services according to patient needs and provides both optical and non- optical devices:

*"Rehabilitation gives you the tools, for example, it introduces you to apps and other technologies. In my case, I have trouble identifying colors and I've been using apps to help me with that. I also use a telescope to help me identify landmarks when I go out alone" (Female, 22 years old, acquired).*

Implementing physical adaptations in public spaces and transportation systems to accommodate autonomous mobility, along with organizational restructuring to align work or academic performance with individual capabilities, represents a promising avenue for enhancing accessibility and inclusivity:

*“I realized that acknowledging my disability at the university was an advantage as it allowed me to organize my semester’s workload based on my ability to handle it. The professors also sought strategies or tools to help me throughout the process” (Female, 22 years old, acquired).*

The preceding information is summarized in the model illustrated in Figure 1, which seeks to conceptualize the stigmatization process from a socio-ecological perspective in the context of health. The process can be broken down into a series of factors, classified as drivers or facilitators, effects, and management. Drivers are inherently negative and comprise a range of elements, including a lack of awareness about low vision, the fragmentation of health services, and the aesthetic characteristics of support devices.

Once stigma is applied, the subsequent effects are observed and involve experiences or practices, such as beliefs, attitudes, and actions. Stigmatizing experiences may include discrimination and the conjunction of adverse social experiences and practices that contribute to internalized stigma, also known as "self-stigma." Finally, the strategies that interviewees identified as crucial for reducing stigmatization are presented.

**Source: Adapted:**
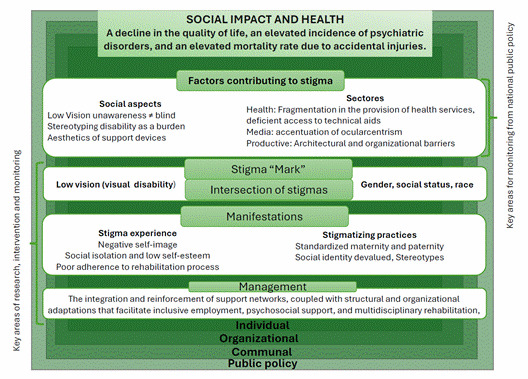
*from Anne L. Stangl et al*^14^

## Discussion

The research shows that individuals with low vision find themselves in an ambivalent position, as they are not recognized as either sighted or blind. This lack of clear identification with a specific group threatens their identity, as they are uncertain about the definition of this "new" version of themselves, which makes it difficult for them to navigate daily activities^22^.

Furthermore, as described by Erving Goffman, individuals with low vision experience a non-visible stigma that can result in a shift in status from "normal" to "discredited." This shift occurs when they rely on devices or practices perceived as abnormal to access visual information^23^. As evidenced in the interviews, this status change may be linked in some cases to difficulty accepting visual loss and reluctance to adopt strategies that favor the use of remaining vision and encourage social participation^24^. In this regard, the vulnerability of individuals using assistive devices must be acknowledged, as “defect-centered care” may potentially infringe on their privacy^25^.

It is important to consider that users of assistive technology may experience feelings of stigmatization due to the social acceptance and aesthetic implications of these devices. In some cases, these feelings may even threaten their lifestyle, leading to an early cessation of use^25^^, ^^26^. Conversely, within the familial and social context, the outward manifestation of visual impairment is often perceived as a sign of helplessness, particularly in maintaining and performing social roles. This perception is further reinforced by phenomena such as ocularcentrism, where vision is privileged^27^, and processes of labeling, stereotyping, segregation, and discrimination are facilitated. These responses can vary considerably depending on the time elapsed since diagnosis^28^.

In conclusion, exploring strategies to mitigate stigma revealed that interaction with other individuals experiencing similar conditions and psychosocial support are conducive to coping. Additionally, educational campaigns, face-to-face interactions, and personal contact have been identified as effective measures, as reported in the literature^22^.

In light of the preceding considerations, it becomes evident that the findings presented here offer a comprehensive and nuanced understanding, at different levels, of how stigma is generated, perpetuated, and addressed among individuals with low vision. This information may prove useful in designing interventions at the individual level. Such interventions might include the development of programs that encourage a positive and resilient self-image in response to stigma while sensitizing and educating family and social support networks to promote attitudes of understanding. These attitudes could support effective inclusion processes in both educational and work environments. Similarly, there is a need to reinforce inclusive policies that promote equal opportunities and encourage positive representation of visual impairment in the media.

The use of interpretative phenomenology is identified as a significant strength of this research, as it facilitated an in-depth exploration of the experiences of individuals with low vision. However, it is acknowledged that including only those who were active at work or academia within the last year may be a limitation, as the stigmatization process may differ considerably for those with lower levels of activity in their daily lives. Furthermore, gender and life cycle perspectives were not considered.

## Conclusion

The stigma associated with low vision is a complex phenomenon influenced by several intrinsic and extrinsic factors. These factors condition the development of stigma awareness, which can affect behavior and psychological well-being.

Awareness of others' prejudices towards individuals with low vision can impact their autonomy, general well-being, and self-esteem. In some instances, it may lead individuals to judge themselves negatively. Furthermore, it can heighten stress and anxiety, as they frequently are on high alert for potential negative attitudes.

Provision of comprehensive care for patients with low vision should extend beyond the scope of traditional medical practice. Instead, it should contribute to the consolidation of support networks that have a positive impact on the quality of life of these patients.
